# Genetic loci associated with changes in lipid levels leading to constitution-based discrepancy in Koreans

**DOI:** 10.1186/1472-6882-14-230

**Published:** 2014-07-09

**Authors:** Sun-Ku Chung, Hyunjoo Yu, Ah Yeon Park, Jong Yeol Kim, Seongwon Cha

**Affiliations:** 1 KM Health Technology Research Group, Medical Research Division, Korea Institute of Oriental Medicine, 1672 Yuseongdae-ro, Yuseong-gu, Daejeon 305-811, Republic of Korea; 2 Medical Engineering R&D Group, Medical Research Division, Korea Institute of Oriental Medicine, 1672 Yuseongdae-ro, Yuseong-gu, Daejeon 305-811, Republic of Korea

**Keywords:** Lipid-associated variants, Cholesterol, Dyslipidemia, *ANGPTL3*, *APOA5-APOA4-APOC3-APOA1*, *APOE-APOC1-APOC4*, *LIPG*, *LPL*, Constitutional type

## Abstract

**Background:**

Abnormal lipid concentrations are risk factors for atherosclerosis and cardiovascular disease. The pathological susceptibility to cardiovascular disease risks such as metabolic syndrome, diabetes mellitus, hypertension, insulin resistance, and so on differs between Sasang constitutional types.

**Methods:**

We used multiple regression analyses to study the association between lipid-related traits and genetic variants from several genome-wide association studies according to Sasang constitutional types, considering that the Tae-Eum (TE) has predominant cardiovascular risk.

**Results:**

By analyzing 26 variants of 20 loci in two Korean populations (8,597 subjects), we found that 12 and 5 variants, respectively, were replicably associated with lipid levels and dyslipidemia risk. By analyzing TE and non-TE type (each 2,664 subjects) populations classified on the basis of Sasang constitutional medicine, we found that the minor allele effects of three variants enriched in TE type had a harmful influence on lipid risk (near apolipoprotein A-V (*APOA5*)*-APOA4-APOC3-APOA1* on increased triglyceride: *p =* 8.90 × 10^-11^, in *APOE-APOC1-APOC4* on increased low-density lipoprotein cholesterol: *p =* 1.63 × 10^-5^, and near endothelial lipase gene on decreased high-density lipoprotein cholesterol: *p =* 4.28 × 10^-3^), whereas those of three variants (near angiopoietin-like 3 gene, *APOA5-APOA4-APOC3-APOA1*, and near lipoprotein lipase gene on triglyceride and high-density lipoprotein cholesterol) associated in non-TE type had neutral influences because of a compensating effect.

**Conclusions:**

These results implied that the minor allele effects of lipid-associated variants may predispose TE type subjects to high cardiovascular disease risk because of their genetic susceptibility to lipid-related disorders.

## Background

Dyslipidemia is characterized by abnormal levels of lipids, including low-density lipoprotein cholesterol (LDLC), high-density lipoprotein cholesterol (HDLC), and triglycerides (TG), in the bloodstream. Although dyslipidemia is not an apparent subjective symptom, it can be a major risk factor for atherosclerosis and cardiovascular disease [1].

Sasang constitutional medicine classified human beings into 4 groups on the basis of distinctive physical, physiological, and psychological characteristics as constitutional types [2]: Tae-Yang, So-Yang, Tae-Eum (TE), and So-Eum. The TE type is associated with a square-shaped face, larger waist circumference, and more sweating in comparison to the other types [3-5]. The So-Eum type is inclined to a slimmer body shape, lower appetite, and increased harm avoidance [4,6-8]. The So-Yang type tends to more physical activity and novelty seeking [7,8]. No more than 0.1% of the Korean population is composed of the Tae-Yang type, which is slender-waisted and more charismatic, although there have been too few population studies to verify this type [9]. Because of the distinctive characteristics mentioned above, the pathological susceptibility to cardiometabolic disorders such as metabolic syndrome, which includes an increased prevalence of dyslipidemia, diabetes mellitus, hypertension, and insulin resistance, also differs between TE and the other types [2,10-13]. Since the Sasang constitutional types are inheritable as revealed by twin and family studies [2], elucidating how the genetic variants can contribute to dyslipidemia susceptibility between constitutional types is necessary, such as several genetic variants associated with obesity- and lipid-related traits of the TE type [14,15].

Several research groups have analyzed the results of genome-wide association studies (GWAS) and replication studies and reported several single nucleotide polymorphisms (SNPs) that affect blood lipid levels and are risk factors for cardiovascular disease [16-19]. Common variants primarily associated with LDLC, HDLC, and TG belong to the following loci: *ABCG5*, *ABCG8*, *SORT1*, *DNAH11*, *HMGCR*, *HNF1A*, *LDLR*, *MAFB*, *PCSK9*, and *TIMD4-HAVCR1*; *ABCA1*, *ANGPTL4*, *CETP*, *GALN2*, *HNF4A*, *LCAT*, *LIPG*, *MADD-FOLH1*, *MVK-MMAB*, and *TTC39B*; and *ANGPTL3*, *GCKR*, *MLXIPL*, *TRIB1*, and *XKR6-AMAC1L2*, respectively. Moreover, some loci associated with TG are simultaneously related to the loci associated with HDLC (5 loci: *APOA5-APOA4-APOC3-APOA1*, *FADS1-FADS2-FADS3*, *LIPC*, *LPL*, and *PLTP*) or LDLC (3 loci: *APOB*, *APOE-APOC* clusters, and *NCAN*).

In this study, we identified SNPs associated with lipid-related traits in TE and non-TE (NTE) type subjects. We also analyzed the genetic risk for lipid disorders to determine which constitutional types are more susceptible to risk for cardiovascular disease.

## Methods

### Subjects

The 5,537 subjects (2,653 men and 2,884 women) were recruited for a population-based cohort study, conducted as part of the Korean Genome and Epidemiology Study (KoGES) for 4 years since 2009 [20], and the 3,060 subjects (1,123 men and 1,937 women) were recruited from 22 oriental medical clinics who were a part of the Korea Constitution Multicenter Study (KCMS) for 6 years since 2006. Of these subjects, 143 KoGES and 1,072 KCMS subjects had disease(s) such as hypertension, dyslipidemia, and/or diabetes. None of the subjects from KoGES and KCMS populations had a history of cancer treatment, thyroid dysfunctions, and postmenopausal hormonal therapy. The recruited individuals were analyzed using an integrated diagnostic model consisting of face, body shape, voice, and questionnaire information [21], namely the Sasang Constitutional Analysis Tool (SCAT), in order to provide a basis for discriminating Sasang constitutional types on the basis of probability values for each constitutional type. On the basis of tertiles of the SCAT probability values for the TE type, we divided all subjects into 3 subgroups. We designated the subjects on the top tertile as TE types (n = 1,824 in the KoGES; n = 840 in the KCMS), and the subjects on the bottom tertile as NTE types (n = 1,824 in the KoGES; n = 840 in the KCMS). All subjects provided written informed consent to participate in the study, and the studies were approved by the Institutional Review Boards of Korea Centers for Disease Control and Prevention (for KoGES) and Korea Institute of Oriental Medicine (for KCMS).

### Selection and genotyping of SNPs

We selected 26 SNPs located in 20 genetic loci from 82 SNPs associated with lipid parameters reported in previous GWASs [16-19]. The 56 SNPs with the following characteristics were excluded (1) 22 SNPs that had low minor allele frequency (under 0.1), (2) 15 SNPs in tight linkage disequilibrium (LD; *r*^*2*^ ≥ 0.7 in Asian HapMap populations) via Haploview program (version 4.2) [22], (3) 4 SNPs that deviated from Hardy–Weinberg equilibrium (*p* < 0.01) in the KoGES subjects, and (4) 15 SNPs (containing the proxy SNPs: *r*^*2*^ ≥ 0.8 in Asian HapMap populations) non-existent in the Affymetrix Genome-Wide Human SNP array 5.0 (Affymetrix, Santa Clara, CA).

The genotypes of the 26 SNPs were determined using 2 genotyping methods by using Affymetrix SNP array 5.0 and unlabeled oligonucleotide probes (UOPs) on the given polymorphic nucleotides [23]. The Affymetrix SNP array 5.0 was used in all KoGES subjects [20] and in 919 KCMS subjects [24], and the UOP genotyping method was used in 2,141 KCMS subjects. First, we screened the 15 replicated SNPs in the KoGES population with 3 lipid parameters such as LDLC, HDLC, and TG, in order to confirm the association of GWAS SNPs with lipid parameters in Koreans. Next, we performed genotyping of 15 SNPs in KCMS subjects in order to double-check their associations with 3 lipid parameters in Koreans. The detailed process of genotyping using UOPs for the 15 selected SNPs (oligonucleotide sequences: see Additional file 1) have been described in a previous report [25]. The genotypes of the 14 SNPs except CETP rs9989419 (*p* < 0.01) did not deviate from Hardy–Weinberg equilibrium in the KCMS population. Therefore, we performed association analyses using the 14 SNPs in the KCMS and combined populations.

### Statistical analysis

Chi-squared test was used to determine whether the SNPs deviated from Hardy–Weinberg equilibrium in each population. LD (Lewontin’s *D′ = D/|D*_*max*_*|* and *r*^*2*^) was obtained from Haploview program, version 4.2 (Daly Lab at the Broad Institute, Cambridge, MA) [22]. Multiple linear regression analyses were performed for LDLC, HDLC, and TG, after adjusting for age; sex; physical activity (3 categories); daily food intake (3 categories); and history and medications for hypertension, dyslipidemia, and/or diabetes. The effects of SNPs associated with dyslipidemia risk (dyslipidemia according to the National Cholesterol Education Program Adult Treatment Panel III guidelines [26]: LDLC ≥ 160 mg/dL, TG ≥ 200 mg/dL, total cholesterol ≥ 240 mg/dL, and/or HDLC < 40 mg/dL (<50 mg/dL for women)) were determined using multiple logistic regression analyses with the same adjustment used in the multiple linear regressions. The *p*-values, after Bonferroni correction for multiple comparisons, were considered significant in the combined linear and logistic regression analyses; *p*-values of <0.05 were considered significant in each population analysis (KoGES and KCMS). Combined analysis was performed in a fixed effect model using Comprehensive Meta-Analysis program, version 2.0 (Biostat, Englewood, NJ). The other regression analyses were performed using R, version 2.15.2 (http://www.r-project.org/).

## Results

### Characteristics of recruited subjects

The characteristics of the subjects from the KoGES and KCMS populations are shown in Table 1. Since the average age and sex ratio between KoGES and KCMS subjects were different, we combined the association results for lipid parameters not by directly merging the 2 populations into one but by conducting meta-analyses of the obtained associations in each population. The TE type subjects presented relatively higher body mass index (BMI), waist circumference, blood pressures, glucose level, and lipid parameters such as LDLC, HDLC, and TG, as well as cardiovascular risk factors such as dyslipidemia than those found in NTE type subjects; these findings were consistent with those of previous reports [4,10-13,27]. A majority of the subjects with TE type had BMI in the overweight range (BMI ≥ 25 kg/m^2^): 27.11 (±2.58) for the KoGES population and 26.35 (±2.83) for the KCMS population (Table 1). Because high BMI along with high waist circumference can pose a higher health risk for diseases such as cardiometabolic disorders, including lipid metabolism disorders, we measured the blood lipid levels, including LDLC, HDLC, and TG, of subjects of the TE type in order to estimate the genetic effects of lipid-associated SNPs according to the constitutional types.

**Table 1:** Characteristics of recruited subjects

	**KoGES**			**KCMS**		
**Characteristic**	**All (n = 5537)**	**TE (n = 1824)**	**NTE (n = 1824)**	**All (n = 3060)**	**TE (n = 840)**	**NTE (n = 840)**
Age (y)	60.48 ± 8.54	61.32 ± 8.61	59.53 ± 8.49	48.09 ± 15.69	52.48 ± 16.03	43.56 ± 14.88
Female (%)	52.09	47.64	56.36	63.30	53.93	73.70
Body mass index (kg/m^2^)	24.46 ± 3.08	27.11 ± 2.58	21.94 ± 2.04	23.47 ± 3.33	26.35 ± 2.83	20.74 ± 2.26
Waist circumference (cm)	86.45 ± 8.39	93.28 ± 6.69	79.76 ± 6.29	84.11 ± 9.88	92.47 ± 7.84	75.91 ± 6.94
Total cholesterol (mg/dL)	193.25 ± 35.16	193.03 ± 34.90	193.90 ± 35.07	186.40 ± 35.33	191.32 ± 36.69	180.30 ± 33.14
LDL cholesterol (mg/dL)	119.96 ± 32.94	120.00 ± 33.35	120.16 ± 33.17	108.29 ± 30.93	113.92 ± 31.43	102.00 ± 28.87
HDL cholesterol (mg/dL)	45.85 ± 12.16	43.98 ± 10.79	47.98 ± 13.32	47.20 ± 12.32	43.30 ± 10.54	52.36 ± 12.65
Triglyceride (mg/dL)	143.28 ± 97.67	156.72 ± 102.30	133.36 ± 101.29	126.23 ± 82.59	153.45 ± 91.97	95.79 ± 57.79
Systolic blood pressure (mmHg)	119.97 ± 16.70	123.22 ± 15.85	116.57 ± 16.81	119.78 ± 15.68	125.44 ± 15.16	113.93 ± 15.00
Diastolic blood pressure (mmHg)	77.65 ± 10.25	79.48 ± 9.95	75.61 ± 10.17	77.10 ± 11.26	80.55 ± 10.71	73.39 ± 11.09
Fasting blood glucose (mg/dL)	101.26 ± 25.16	104.74 ± 27.10	97.75 ± 21.98	98.73 ± 28.24	106.26 ± 34.89	93.16 ± 17.89
Dyslipidemia (%)	62.96	69.96	59.65	57.06	70.36	40.60

Data are given as mean ± standard deviation or %.

Dyslipidemia: LDLC ≥ 160 mg/dL, TG ≥ 200 mg/dL, TC ≥ 240 mg/dL, and/or HDLC < 40 mg/dL (<50 mg/dL for women).

### Lipid-associated SNPs according to constitutional types

We reanalyzed the genome-wide associations of 26 SNPs located in 20 gene loci selected from previous reports [16-19] with lipid parameters, including LDLC, HDLC, and TG, in the 2 populations. Of the 15 SNPs (SNP list: see Additional file 1) showing associations with the lipid parameters in the KoGES population, 12 in 10 gene loci were selected from the analyses with a significance of *p <* 0.00192 by Bonferroni correction (0.05 divided by 26 SNPs) in the combined population (Table 2). The association signals of 3 SNPs, rs10889353, rs2156552, and rs17321515, were not significant in the KCMS population, although the combined association signals were significant. The lipid phenotypes associated with the 12 SNPs well corresponded with the associations reported in the previous papers [16-19]. The most significant SNP was rs6589566 located near apolipoprotein A-V (*APOA5*) gene and exerted an effect on TG levels in both populations (KoGES, *p* < 2.0 × 10^-16^; KCMS, *p* = 7.25 × 10^-12^). Among the SNPs that showed an overlap between lipid traits and associated SNPs, rs4149270 (ATP-binding cassette transporter A1 (*ABCA1*)), rs6589566 (*APOA5*), rs4420638 (apolipoprotein C-I (*APOC1*)), rs261332 (hepatic lipase (*LIPC*)), rs2156552 (endothelial lipase (*LIPG*)), and 2 SNPs of lipoprotein lipase (*LPL*), rs1919484 and rs6586891, were found to be associated with HDLC (combined *p*-value: 3.10 × 10^-7^, 1.22 × 10^-5^, 8.87 × 10^-5^, 1.90 × 10^-8^, 2.61 × 10^-4^, 1.45 × 10^-6^, and 9.39 × 10^-9^, respectively). For TG, associations were found among rs10889353 and rs1748195 (angiopoietin-like 3 (*ANGPTL3*)), rs6589566 (*APOA5*), rs4420638 (*APOAC1*), rs174547 (fatty acid desaturase 1 (*FADS1*)), rs261332 (*LIPC*), and rs1919484 and rs6586891 (*LPL*) (combined *p*-value: 3.25 × 10^-5^, 6.28 × 10^-5^, <2.0 × 10^-16^, 4.80 × 10^-4^, 6.25 × 10^-5^, 8.84 × 10^-4^, 3.68 × 10^-6^, and 5.56 × 10^-6^, respectively). Since the SNPs of the *ANGPTL3* and *LPL* genes were in strong LD within each locus in the KCMS population (*r*^*2*^ = 0.72, *D′* = 0.99 for *ANGPTL3* SNPs; *r*^*2*^ = 0.40, *D′* = 0.87 for *LPL* SNPs), the association patterns were similar between the 2 SNPs in each locus. Therefore, we only focused on the effects of rs10889353 for the *ANGPTL3* allele and rs6586891 for the *LPL* allele in the following analyses, which showed higher significant signals in the combined analyses. For additional LDLC, we selected rs4420638 and rs3846663 located near *APOC1* and 3-hydroxy-3-methylglutaryl-CoA-reductase (*HMGCR*) gene loci, respectively (combined *p*-value: 4.87 × 10^-8^ and 8.75 × 10^-5^, respectively).

**Table 2:** Linear regression analysis for lipid traits in the Korean population

			**Combined**^**†**^			**KoGES**			**KCMS**		
**SNP (allele, gene)**	**MAF**	Trait*	**n**	**Effect (SE) [mg/dL]**	***P*-value**^**‡**^	**n**	**Effect (SE) [mg/dL]**	***P*-value**^**‡**^	**n**	**Effect (SE) [mg/dL]**	***P*-value**^**‡**^
rs4149270 (C>T, *ABCA1*)	0.348	HDLC	**8554**	**-0.956 (0.187)**	**3.10 × 10**^**-7**^	**5528**	**-0.806 (0.236)**	**6.51 × 10**^**-4**^	**3026**	**-1.205 (0.305)**	**7.93 × 10**^**-5**^
rs10889353 (A>C, *ANGPTL3*)	0.173	TG	8575	-0.018 (0.004)	3.25 × 10^-5^	5536	-0.021 (0.005)	1.18 × 10^-4^	3039	-0.013 (0.007)	0.0739
rs1748195 (C>G, *ANGPTL3*)	0.221	TG	**8582**	**-0.016 (0.004)**	**6.28 × 10**^**-5**^	**5536**	**-0.018 (0.005)**	**4.83 × 10**^**-4**^	**3046**	**-0.013 (0.007)**	**4.36 × 10**^**-2**^
rs6589566 (A>G, *APOA5*)	0.217	TG	**8569**	**0.044 (0.004)**	**< 2.0 × 10**^**-16**^	**5528**	**0.043 (0.005)**	**< 2.0 × 10**^**-16**^	**3041**	**0.046 (0.007)**	**7.25 × 10**^**-12**^
		HDLC	**8569**	**-0.963 (0.220)**	**1.22 × 10**^**-5**^	**5528**	**-0.935 (0.278)**	**7.65 × 10**^**-4**^	**3041**	**-1.009 (0.361)**	**5.19 × 10**^**-3**^
rs4420638 (T>C, *APOC1*)	0.115	LDLC	**8579**	**4.103 (0.752)**	**4.87 × 10**^**-8**^	**5528**	**4.287 (0.951)**	**6.61 × 10**^**-6**^	**3051**	**3.796 (1.230)**	**2.04 × 10**^**-3**^
		TG	**8579**	**0.018 (0.005)**	**4.80 × 10**^**-4**^	**5528**	**0.016 (0.0006)**	**1.47 × 10**^**-2**^	**3051**	**0.023 (0.009)**	**1.01 × 10**^**-2**^
		HDLC	**8579**	**-1.108 (0.283)**	**8.87 × 10**^**-5**^	**5528**	**-0.983 (0.353)**	**5.41 × 10**^**-3**^	**3051**	**-1.329 (0.283)**	**4.80 × 10**^**-3**^
rs174547 (T>C, *FADS1*)	0.330	TG	**8575**	**0.014 (0.004)**	**6.25 × 10**^**-5**^	**5533**	**0.014 (0.004)**	**1.45 × 10**^**-3**^	**3042**	**0.014 (0.006)**	**1.54 × 10**^**-2**^
rs3846663 (T>C, *HMGCR*)	0.473	LDLC	**8587**	**-1.871 (0.477)**	**8.75 × 10**^**-5**^	**5537**	**-1.872 (0.611)**	**2.21 × 10**^**-3**^	**3050**	**-1.871 (0.763)**	**1.42 × 10**^**-2**^
rs261332 (G>A, *LIPC*)	0.215	TG	**8578**	**0.014 (0.004)**	**8.84 × 10**^**-4**^	**5528**	**0.012 (0.005)**	**1.51 × 10**^**-2**^	**3050**	**0.015 (0.007)**	**2.17 × 10**^**-2**^
		HDLC	**8578**	**1.233 (0.219)**	**1.90 × 10**^**-8**^	**5528**	**1.124 (0.278)**	**5.36 × 10**^**-5**^	**3050**	**1.413 (0.357)**	**7.78 × 10**^**-5**^
rs2156552 (A>T, *LIPG*)	0.164	HDLC	8583	-0.884 (0.242)	2.61 × 10^-4^	5536	-1.019 (0.309)	9.84 × 10^-4^	3047	-0.670 (0.390)	0.0858
rs1919484 (G>A, *LPL*)	0.208	TG	**8577**	**-0.019 (0.004)**	**3.68 × 10**^**-6**^	**5535**	**-0.016 (0.005)**	**2.12 × 10**^**-3**^	**3042**	**-0.025 (0.007)**	**3.02 × 10**^**-4**^
		HDLC	**8577**	**1.062 (0.220)**	**1.45 × 10**^**-6**^	**5535**	**0.733 (0.277)**	**8.04 × 10**^**-3**^	**3042**	**1.633 (0.365)**	**7.85 × 10**^**-6**^
rs6586891 (C>A, *LPL*)	0.325	TG	**8579**	**-0.016 (0.004)**	**5.56 × 10**^**-6**^	**5536**	**-0.015 (0.004)**	**7.91 × 10**^**-4**^	**3043**	**-0.018 (0.006)**	**2.04 × 10**^**-3**^
		HDLC	**8579**	**1.096 (0.191)**	**9.39 × 10**^**-9**^	**5536**	**1.031 (0.242)**	**2.13 × 10**^**-5**^	**3043**	**1.203 (0.310)**	**1.07 × 10**^**-4**^
rs17321515 (G>A, *TRIB1*)	0.439	TG	8573	0.013 (0.003)	1.59 × 10^-4^	5528	0.015 (0.0004)	3.19 × 10^-4^	3045	0.008 (0.006)	0.136

*Triglyceride (TG): log-transformed.

^†^Combined: combined meta-analysis of association signals in the KoGES and KCMS populations in a fixed effect model.

^‡^Superscript *p*-values indicate significant associations. Significance: *p <* 0.00192 (0.05/26 SNPs) in the combined population; *p <* 0.05 in the KoGES and KCMS populations.

Boldface letters indicate reproducible associations in all three populations.

To determine genetic discrepancy for cardiovascular risks between TE and NTE types, we extracted 2 subgroups, TE and NTE types, from all subjects, on the basis of the tertiles of the SCAT probability values for TE constitutional types [21]: TE type, in the top tertile, presented a constitutional type with high cardiovascular risk, and NTE type, in the bottom tertile, presented a constitutional type with low cardiovascular risk. Of the 10 lipid-associated SNPs of 10 loci in all population, 5 showed significant and reproducible associations with lipid parameters in TE and/or NTE types (Table 3): the minor allele effects of 3 SNPs (rs6589566 on TG, rs4420638 on LDLC, and rs2156552 on HDLC) were enriched in TE type, whereas those of 3 SNPs (rs10889353 on TG, rs6589566 on HDLC, and rs6586891 on HDLC) were enriched in NTE type. The TG-increasing effect of rs6589566 was not enriched in NTE type (the effect in NTE type was smaller than that in all subjects), but remained significant.

**Table 3:** Linear regression analysis for lipid traits in the subgroups of constitutional types

			**Combined**^**†**^			**KoGES**			**KCMS**		
**SNP (allele, gene)**	Trait*	**Subject**	**n**	**Effect (SE) [mg/dL]**	***P*-value**^**‡**^	**n**	**Effect (SE) [mg/dL]**	***P*-value**^**‡**^	**n**	**Effect (SE) [mg/dL]**	***P*-value**^**‡**^
rs4149270	HDLC	TE	2646	-0.717 (0.301)	0.0171	1821	-0.728 (0.371)	4.97 × 10^-2^	825	-0.695 (0.514)	0.177
(C>T, *ABCA1*)		NTE	2658	-1.144 (0.367)	1.82 × 10^-3^	1822	-1.173 (0.453)	9.60 × 10^-3^	836	-1.088 (0.627)	0.0802
rs10889353	TG	TE	2656	-0.018 (0.008)	0.0233	1823	-0.020 (0.010)	4.35 × 10^-2^	833	-0.015 (0.014)	0.286
(A>C, *ANGPTL3*)		**NTE**	**2658**	**-0.023 (0.008)**	**2.33 × 10**^**-3**^	**1824**	**-0.022 (0.010)**	**2.35 × 10**^**-2**^	**834**	**-0.026 (0.013)**	**4.04 × 10**^**-2**^
rs6589566	TG	**TE**	**2656**	**0.047 (0.007)**	**8.90 × 10**^**-11**^	**1820**	**0.047 (0.009)**	**2.09 × 10**^**-7**^	**836**	**0.049 (0.013)**	**1.22 × 10**^**-4**^
(A>G, *APOA5*)		**NTE**	**2654**	**0.034 (0.007)**	**3.09 × 10**^**-6**^	**1823**	**0.030 (0.009)**	**1.04 × 10**^**-3**^	**831**	**0.042 (0.012)**	**6.99 × 10**^**-4**^
	HDLC	TE	2656	-0.655 (0.350)	0.0615	1820	-0.698 (0.436)	0.110	836	-0.577 (0.589)	0.328
		**NTE**	**2654**	**-1.519 (0.440)**	**5.55 × 10**^**-4**^	**1823**	**-1.381 (0.539)**	**1.05 × 10**^**-2**^	**831**	**-1.795 (0.762)**	**1.87 × 10**^**-2**^
rs4420638	LDLC	**TE**	**2659**	**6.028 (1.399)**	**1.63 × 10**^**-5**^	**1821**	**5.713 (1.731)**	**9.86 × 10**^**-4**^	**838**	**6.619 (2.373)**	**5.40 × 10**^**-4**^
(T>C, *APOC1*)		NTE	2654	2.789 (1.334)	0.0365	1820	3.027 (1.662)	0.0687	834	2.358 (2.236)	0.292
rs261332	HDLC	TE	2658	1.026 (0.349)	3.29 × 10^-3^	1820	0.573 (0.436)	0.189	838	1.835 (0.583)	1.70 × 10^-3^
(G>A, *LIPC*)		NTE	2655	1.931 (0.434)	8.51 × 10^-6^	1821	2.204 (0.529)	3.21 × 10^-5^	834	1.369 (0.758)	0.0713
rs2156552	HDLC	**TE**	**2660**	**-1.102 (0.386)**	**4.28 × 10**^**-3**^	**1823**	**-0.961 (0.477)**	**4.41 × 10**^**-2**^	**837**	**-1.370 (0.656)**	**3.72 × 10**^**-2**^
(A>T, *LIPG*)		NTE	2657	-1.049 (0.478)	0.0283	1824	-1.383 (0.596)	2.03 × 10^-2^	833	-0.441 (0.804)	0.583
rs6586891	TG	TE	2661	-0.015 (0.006)	0.0229	1824	-0.012 (0.008)	0.131	837	-0.021 (0.011)	0.0685
(C>A, *LPL*)		NTE	2656	-0.018 (0.006)	3.38 × 10^-3^	1824	-0.017 (0.008)	2.81 × 10^-2^	832	-0.020 (0.010)	0.0517
	HDLC	TE	2661	0.897 (0.309)	3.71 × 10^-3^	1824	0.718 (0.379)	0.0584	837	1.251 (0.534)	1.93 × 10^-2^
		**NTE**	**2656**	**1.404 (0.378)**	**2.00 × 10**^**-4**^	**1824**	**1.046 (0.472)**	**2.68 × 10**^**-2**^	**832**	**2.041 (0.629)**	**1.23 × 10**^**-3**^

*Triglyceride (TG): log-transformed.

^†^Combined: combined meta-analysis of association signals in the KoGES and KCMS populations in a fixed effect model.

^‡^Superscript *p*-values indicate significant associations. Significance: *p <* 0.005 (0.05/10 SNPs) in the combined population; *p <* 0.05 in the KoGES and KCMS populations.

Boldface letters indicate reproducible associations in all three populations.

In addition, by analyzing the associations of the 10 lipid-associated SNPs with dyslipidemia risk, we found that 5 SNPs, rs4149270, rs6589566, rs4420638, rs261332, and rs6586891, were significantly associated in the combined population (*p* < 0.005; Bonferroni correction, 0.05/10 SNPs) (combined *p*-value: 1.55 × 10^-5^ for rs4149270, 2.77 × 10^-4^ for rs6589566, 0.00218 for rs261332, and 4.31 × 10^-6^ for rs6586891) (Table 4). The 3 SNPs, rs4149270, rs6589566, and rs6586891, were repeatedly associated with dyslipidemia in both the populations, but the other 2 SNPs were not significant in one of the 2 populations. After the subgroup analysis, only rs261332 of the *LIPC* gene was significantly associated with dyslipidemia in the combined NTE type, but the association was not reproduced in the KCMS population (Table 4). In contrast, *LPL* rs6586891 was reproducibly associated in the NTE types of the KoGES and KCMS populations, but was not significant in the combined analysis of the NTE type.

**Table 4:** Logistic regression analysis for dyslipidemia risk

		Combined*		**KoGES**				**KCMS**			
				**n (MAF)**				**n (MAF)**			
**SNP (allele, gene)**	**Subject**	**OR (SE)**	***P*-value**^**†**^	**Control**	**Case**	**OR (SE)**	***P*-value**^**†**^	**Control**	**Case**	**OR (SE)**	***P*-value**^**†**^
rs4149270 (C>T, *ABCA1*)	**All**	**1.157 (0.034)**	**1.55 × 10**^**-5**^	**2048 (0.330)**	**3480 (0.359)**	**1.136 (0.042)**	**2.49 × 10**^**-3**^	**1305 (0.275)**	**1728 (0.366)**	**1.194 (0.056)**	**1.55 × 10**^**-3**^
	TE	1.185 (0.064)	0.00826	594 (0.305)	1227 (0.350)	1.199 (0.077)	1.84 × 10^-2^	242 (0.349)	583 (0.387)	1.151 (0.115)	0.222
	NTE	1.126 (0.060)	0.0491	755 (0.342)	1067 (0.364)	1.135 (0.073)	0.0816	499 (0.327)	337 (0.347)	1.106 (0.106)	0.344
rs6589566 (A>G, *APOA5*)	**All**	**1.156 (0.040)**	**2.77 × 10**^**-4**^	**2047 (0.203)**	**3481 (0.224)**	**1.150 (0.050)**	**5.13 × 10**^**-3**^	**1305 (0.203)**	**1735 (0.228)**	**1.167 (0.066)**	**1.99 × 10**^**-2**^
	TE	1.148 (0.075)	0.0648	593 (0.205)	1227 (0.222)	1.128 (0.090)	0.182	247 (0.206)	589 (0.235)	1.195 (0.134)	0.185
	NTE	1.222 (0.072)	0.00536	755 (0.203)	1068 (0.224)	1.176 (0.087)	0.0628	493 (0.195)	338 (0.235)	1.328 (0.128)	2.68 × 10^-2^
rs4420638 (T>C, *APOC1*)	All	1.232 (0.052)	5.53 × 10^-5^	2050 (0.100)	3478 (0.125)	1.286 (0.065)	9.52 × 10^-5^	1309 (0.107)	1742 (0.120)	1.140 (0.087)	0.132
	TE	1.158 (0.100)	0.142	593 (0.094)	1227 (0.115)	1.266 (0.122)	0.0528	248 (0.113)	590 (0.114)	0.962 (0.176)	0.828
	NTE	1.186 (0.091)	0.0614	755 (0.109)	1065 (0.131)	1.220 (0.109)	0.0677	495 (0.103)	339 (0.111)	1.110 (0.168)	0.535
rs261332(G>A, *LIPC*)	All	0.883 (0.049)	2.18 × 10^-3^	2047 (0.226)	3481 (0.214)	0.918 (0.049)	0.0823	1308 (0.225)	1745 (0.198)	0.834 (0.065)	5.38 × 10^-3^
	TE	0.901 (0.082)	0.177	591 (0.222)	1229 (0.216)	0.959 (0.089)	0.635	248 (0.242)	590 (0.204)	0.807 (0.128)	0.0934
	NTE	0.818 (0.071)	4.68 × 10^-3^	755 (0.232)	1066 (0.201)	0.810 (0.085)	1.27 × 10^-2^	495 (0.211)	339 (0.187)	0.839 (0.129)	0.175
rs6586891 (C>A, *LPL*)	**All**	**0.854 (0.034)**	**4.31 × 10**^**-6**^	**2051 (0.343)**	**3485 (0.315)**	**0.859 (0.043)**	**4.37 × 10**^**-4**^	**1304 (0.345)**	**1739 (0.309)**	**0.846 (0.057)**	**3.10 × 10**^**-3**^
	TE	0.867 (0.065)	0.0275	594 (0.329)	1230 (0.316)	0.919 (0.078)	0.276	247 (0.360)	590 (0.302)	0.758 (0.118)	0.190
	NTE	0.842 (0.062)	0.00555	755 (0.341)	1069 (0.313)	0.862 (0.076)	4.88 × 10^-2^	494 (0.333)	338 (0.288)	0.803 (0.108)	4.32 × 10^-2^

*Combined: combined meta-analysis of association signals in the KoGES and KCMS populations in a fixed effect model.

^†^Superscript *p*-values indicate significant associations. Significance: *p <* 0.005 (0.05/10 SNPs) in the combined population; *p <* 0.05 in the KoGES and KCMS populations.

Boldface letters indicate reproducible associations in all three populations.

## Discussion

Analysis of the polymorphisms of gene loci associated with lipid-related traits revealed significant associations of 12 SNPs out of the 26 selected GWAS SNPs; these SNPs were located on *ABCA1*, *ANGPTL3*, *APOA5-APOA4-APOC3-APOA1*, *APOE-APOC1-APOC4*, *FADS1-FADS2-FADS3*, *HMGCR*, *LIPC*, *LIPG*, *LPL*, and *TRIB1* gene regions. Interestingly, minor alleles of the 3 SNPs of *APOA5-APOA4-APOC3-APOA1*, *APOE-APOC1-APOC4*, and *LIPG* that were enriched in TE type subjects exerted harmful influences on lipid risk, whereas the minor alleles of the SNPs associated in NTE type subjects had both beneficial and harmful, maybe neutral, influences on lipid risk (Table 3). The rs6589566 near *APOA5* significantly affected TG levels in both TE and NTE types, which corresponded with the previously reported association of rs662799 (strong LD with rs6589566: *r*^*2*^ = 0.59, *D′* = 0.97 in the KCMS population) with TG levels in TE type as well as in So-Yang type subjects who partly belonged to the NTE type [15]. For dyslipidemia risk, no significant reproducible SNP associations were noted in the subgroup analysis for the constitutional type. Taken together, the minor allele effects of lipid-associated SNPs appear to reflect the characteristics of both the types (high in TE type and low in other types) with regard to cardiovascular risk [2].

In the studied populations, most of the lipid-associated loci such as *ABCA1*, *APOA1*, *APOE*, *LIPC*, and *LPL* were known to be involved in lipoprotein metabolism [28]. Cholesterol is excreted from peripheral tissues such as intestine, liver, and macrophages, via the ABCA1 membrane transporter to form HDL in association with the lipoprotein, APOA1 [28]. LPL, which can be activated by APOA5 and inhibited by ANGPTL3 [29,30], hydrolyzes TG packaged into chylomicron and very low-density lipoprotein, TG-rich lipoprotein particles, and LIPC is involved in converting intermediate-density lipoproteins formed from very low-density lipoproteins into LDL. In addition, LIPG is also known to stimulate cholesteryl ester uptake through hepatic scavenger receptor class B type I [31], and APOC1 inhibits the action of cholesteryl ester transfer protein [32] that is involved in transferring cholesteryl ester from HDL to intermediate-density lipoproteins. On the basis of this metabolism mechanism, we could speculate the coordinated action of the lipid-associated SNPs in each constitutional type. Since the minor allele effect of *APOA5* rs6589566 was opposite to that of *LPL* rs6586891 on the levels of TG and HDLC, the LPL activity reducing the TG level [29] may be stimulated by the minor allele of rs6586891 and inhibited by the minor allele of rs6589566. In the NTE type (Table 3), the minor alleles of rs4149270 near *ABCA1* and rs6589566 near *APOA1* may reduce the HDLC level, and the reduced inhibitory effect of the minor allele of *ANGPTL3* rs10889353 on the LPL activity may affect the reduction and increment in the levels of TG and HDLC, respectively. On the other hand, the reduced inhibitory effect of the C allele of *APOC1* rs4420638 on the activity of cholesteryl ester transfer protein may increase the LDLC level in TE type, and the HDLC-decreasing effect of *LIPG* rs2156552 minor allele may be exerted because the hepatic uptake of cholesteryl ester is affected (Table 3). Therefore, despite the unknown mechanisms of lipid-associated SNPs, their effects might reflect the relative pathological predisposition of each constitutional type. Even though they are present in the noncoding region and lack proper function, lipid-associated SNPs might affect the expression of the genes harboring them. Indeed, some groups have reported that lipid-associated gene expression depends on distinct minor allelic frequency in the human liver, strongly suggesting that lipid-associated SNPs might influence gene expression as the *cis*-acting regulator at the noncoding region [17].

The reason for the low frequency of lipid-associated SNPs in the studied populations (12 SNPs from 26 SNPs) compared to that in Caucasians might be due to the ethnic differences and small population size to detect SNPs that have a minor role [16-19]. For example, the minor allele frequencies (0.17, 0.12, 0.47, and 0.44, respectively) of rs10889353, rs4420638, rs3846663, and rs17321515 of 12 lipid-associated SNPs in Koreans were different from those of the SNPs in Caucasians (0.32, 0.82, 0.62, and 0.56, respectively).

According to previous reports [4,10-13], TE type subjects are susceptible to diverse metabolic diseases, including obesity, diabetes, and hypertension, which are well-known risk factors of cardiovascular diseases. In addition, dyslipidemia that is characterized by the formation of atherosclerotic plaque in the bloodstream, an additional risk for cardiovascular disease, is also prevalent in TE type [10]. By analyzing the lipid-associated SNPs in the TE and NTE type Koreans, we found that each constitutional type has a distinct profile of SNPs associated with lipid parameters, presenting a genetic predisposition to vascular disease risk, especially in the TE type.

## Conclusions

In conclusion, we found that 12 SNPs (in 10 gene loci) of 26 SNPs (in 20 gene loci) were associated with the genetic risk for lipid-related disorders. The constitution-based genetic profiles for lipid levels were different between TE and NTE types. That is, the TG-elevating *APOA5* minor allele, the LDLC-elevating *APOC1* minor allele, and HDLC-decreasing *LIPG* minor allele were significantly enriched in TE type, whereas minor alleles of 3 SNPs, *ANGPTL3*, *APOA5*, and *LPL*, compensated for the effects on TG and HDLC. Therefore, our findings suggest that TE type, unlike NTE type, may be at a higher risk of cardiovascular diseases because of the genetic risk for lipid-related disorders.

## Abbreviations

ABCA1: ATP-binding cassette transporter A1; ANGPTL3: Angiopoietin-like 3; APOA5: Apolipoprotein A-V; APOC1: Apolipoprotein C-I; BMI: Body mass index; FADS1: Fatty acid desaturase 1; GWAS: Genome-wide association studies; HDLC: High-density lipoprotein cholesterol; HMGCR: 3-hydroxy-3-methylglutaryl-CoA-reductase; KCMS: Korea constitution multicenter study; KoGES: Korean genome and epidemiology study; LDLC: Low-density lipoprotein cholesterol; LIPC: Hepatic lipase; LIPG: Endothelial lipase; LPL: Lipoprotein lipase; MAF: Minor allele frequency; NTE: Non-tae-eum; OR: Odds ratio; SCAT: Sasang constitutional analysis tool; SNPs: Single nucleotide polymorphisms; TE: Tae-eum; TG: Triglycerides; UOPs: Unlabeled oligonucleotide probes.

## Competing interests

The authors declare that they have no competing interests.

## Authors’ contributions

SKC interpreted the data and drafted the manuscript. HY acquired the data, performed the statistical analyses, and helped to draft the manuscript. AYP acquired the data and helped to draft the manuscript. JYK recruited subjects, managed the clinical data, and helped to design the study. SC conceived and designed the study, performed the statistical analyses, interpreted the data, and drafted the manuscript. All authors approved the final manuscript.

## Pre-publication history

The pre-publication history for this paper can be accessed here:

http://www.biomedcentral.com/1472-6882/14/230/prepub

## Supplementary Material

Additional file 1 The oligonucleotide sequences of primers and UOPs for 15 SNPs genotyped in a KCMS population. Click here for file
